# Therapeutic Targeting of Innate Immune Receptors Against SARS-CoV-2 Infection

**DOI:** 10.3389/fphar.2022.915565

**Published:** 2022-06-30

**Authors:** Mariya Farooq, Abdul Waheed Khan, Bilal Ahmad, Moon Suk Kim, Sangdun Choi

**Affiliations:** ^1^ Department of Molecular Science and Technology, Ajou University, Suwon, South Korea; ^2^ S&K Therapeutics, Ajou University, Suwon, South Korea

**Keywords:** SARS-CoV-2, innate immune receptor, toll-like receptor, RIG-like receptor, antiviral response

## Abstract

The innate immune system is the first line of host’s defense against invading pathogens. Multiple cellular sensors that detect viral components can induce innate antiviral immune responses. As a result, interferons and pro-inflammatory cytokines are produced which help in the elimination of invading viruses. Severe acute respiratory syndrome coronavirus 2 (SARS-CoV-2) belongs to Coronaviridae family, and has a single-stranded, positive-sense RNA genome. It can infect multiple hosts; in humans, it is responsible for the novel coronavirus disease 2019 (COVID-19). Successful, timely, and appropriate detection of SARS-CoV-2 can be very important for the early generation of the immune response. Several drugs that target the innate immune receptors as well as other signaling molecules generated during the innate immune response are currently being investigated in clinical trials. In this review, we summarized the current knowledge of the mechanisms underlying host sensing and innate immune responses against SARS-CoV-2 infection, as well as the role of innate immune receptors in terms of their therapeutic potential against SARS-CoV-2. Moreover, we discussed the drugs undergoing clinical trials and the FDA approved drugs against SARS-CoV-2. This review will help in understanding the interactions between SARS-CoV-2 and innate immune receptors and thus will point towards new dimensions for the development of new therapeutics, which can be beneficial in the current pandemic.

## 1 Introduction

Coronaviridae is a family of enveloped positive-sense single-stranded RNA viruses. In the last 19 years, this virus family has been the cause of three different zoonotic outbreaks, and the latest one—caused by severe acute respiratory syndrome coronavirus 2 (SARS-CoV-2)—resulted in the COVID-19 pandemic (Gorbalenya et al., 2020). The family of coronaviridae can be divided into four genera: alpha (*α*), beta (*ß*), gamma (*γ*), and delta (Δ) (Sheahan et al., 2020). *Alpha-* and *Betacoronavirus* members have been reported to infect humans. Being the seventh member of Coronaviridae involved in a zoonosis, SARS-CoV-2 belongs to the genus *Betacoronavirus* (Li X. et al., 2020). With a particle size of 125 nm, this ball-shaped virus has club-shaped appendages; a spike protein embedded into the envelope, giving it a crown-like appearance in an electron micrograph, hence the name coronavirus (Anderson and Schneider, 2012).

The virus was first identified in China, and the World Health Organization (WHO) used a provisional nonspecific name: “2019 novel coronavirus” (2019-nCoV). Later, based on the phylogenetic analysis of the other coronaviruses, the Coronavirus Study Group of the International Committee on Virus Taxonomy suggested renaming it as SARS-CoV-2 (Awadasseid et al., 2020).

Coronaviruses have one of the largest RNA genomes, with a size ranging between 26 and 32 kb. SARS-CoV-2 has a genome length of 30 kbp, and the genome is flanked by 3′ and 5′ untranslated regions and contains 15 open reading frames (ORFs). The genome translates into a polyprotein of approximately 800 kDa. There are 4 structural proteins that are encoded by 2–14 open reading frames: Nucleocapsid (N), envelope (E) and Spike (S) and Membrane protein (M). The structural proteins play their role in virion assembly and infection; and the non-structural proteins are involved in the replication cycle of the virus (Mirza and Froeyen, 2020). The shape of the virus is maintained by the M protein. The M protein binds to Nucleocapsid, which constitutes almost 1% of the expressed protein during infection, and it binds to the viral RNA genome through its two different subunits. It helps to package the genome inside the virion (Chen Y. et al., 2020; Jackson et al., 2021a; Tugaeva et al., 2021). E and M interact to form the envelope of the virus. Receptor Binding Domain (RBD) of Spike, that is glycoprotein in nature, is responsible for interaction between virus and the host cell receptors thus helps the virus to enter the cell (Luan et al., 2020).

The first two ORFs encode nonstructural proteins. There are 16 of these proteins, which are Nsp1 through Nsp16. Nsp1 (protein ID: YP_009725297.1) has a length of 180 amino acid residues (aa). Nsp2 (YP_009725298.1) consists of 638 aa. Nsp3 (YP_009725299.1) consists of 1945 aa. It contains the following domains: N-terminal acidic (Ac), predicted phosphoesterase, papain-like proteinase, Y-domain, transmembrane domain 1 (TM1), and adenosine diphosphate-ribose 1″-phosphatase (ADRP). Nsp4 (YP_009725300.1) has a length of 500 aa and contains transmembrane domain 2. Nsp5 (YP_009725301.1) is 3C-like proteinase (3Cl-pro), and its length is 306 aa. Nsp6 (protein ID YP_009725302.1) is a putative transmembrane domain having a length of 290 aa. Nsp7 (YP_009725303.1) and Nsp8 (YP_009725304.1) are proposed cofactors for the polymerase. Lengths of their genes are 249 and 339 nucleotides encoding an 83 aa protein and 198 aa proteins, respectively. Nsp9 (YP_009725305.1) is a single-stranded RNA (ssRNA)-binding protein whose length is 113 aa. Nsp10 (YP_009725306.1) has a length of 139 aa and is also called CysHis; it has previously been known as growth factor-like protein (GFL). The length of Nsp11 (YP_009725295.1) is 13 aa. Nsp12 (YP_009725307.1), with a length of 932 aa, carries an RNA-dependent RNA polymerase (RdRp) domain and NIRAN domain. Nsp13 (YP_009725308.1) is helicase, which contains a zinc-binding domain (NTPase/helicase domain (HEL)) and RNA 5′-triphosphatase domain and has a length of 601 aa. Nsp14 (YP_009725309.1) is 3′-to-5′ exonuclease and has a length of 521 aa. Nsp15 (YP_009725310.1), with a length of 346 aa, is endoRNase. Nsp16 (YP_009725311) is 2′-O-ribose methyltransferase and is 298 aa long. Structural and nonstructural proteins and their details are summarized in Tables 1 and 2 (extracted from https://www.ncbi.nlm.nih.gov/gene/43740578).

**TABLE 1:** Summary of the Non-structural proteins.

Sr No.	Protein	GenBank ID	Start point (nt)	End point (nt)	Length (nt)	Length (aa)	MW (kDa)	Crystal Structure	Stability	Function
1	Nsp1	YP_009725297.1	266	805	540	180	19.9	No	Stable	Leader protein
2	Nsp2	YP_009725298.1	806	2719	1914	639	70.5	No	Stable	Disruption of the host cell environment
3	Nsp3	YP_009725299.1	2720	8554	5835	1935	217.2	Yes	Stable	Papain Like Protease
4	Nsp4	YP_009725300.1	8555	10054	1500	500	56.2	No	Stable	Contains transmembrane domain 2
5	Nsp5	YP_009725301.1	10055	10972	918	306	33.5	Yes	Stable	3C-like proteinase
6	Nsp6	YP_009725302.1	10973	11842	870	293	33.7	No	Stable	Putative transmembrane domain
7	Nsp7	YP_009725303.1	11843	12091	249	83	92.9	Yes	Unstable	Co-factor of Nsp12
8	Nsp8	YP_009725304.1	12092	12685	339	210	23.2	Yes	Stable	Co-factor of Nsp12
9	Nsp9	YP_009725305.1	12686	13024	339	111	11.9	Yes	Stable	ssRNA-binding protein
10	Nsp10	YP_009725306.1	13025	13441	417	145	15.4	Yes	Stable	CysHis; formerly known as growth-factor-like protein (GFL)
11	Nsp11	YP_009725312.1	13442	13480	39	23	23.7	No	Stable	Not known
12	Nsp12	YP_009725307.1	13442	16236	2696	932	106.7	Yes	Stable	RNA-dependent RNA polymerase
13	Nsp13	YP_009725308.1	16237	19039	1803	601	66.9	Yes	Stable	Helicase
14	Nsp14	YP_009725309.1	18040	19620	1581	527	59.8	No	Stable	3′-to-5′ exonuclease
15	Nsp15	YP_009725310.1	19621	20658	1038	346	38.8	Yes	Stable	EndoRNAse
16	Nsp16	YP_009725311.1	20659	21552	894	298	33.3	Yes	Stable	2′-O-ribose methyltransferase

**TABLE 2:** Summary of the Structural proteins.

Sr. No.	Protein	GenBank ID	Start point (nt)	End point (nt)	Length (nt)	Length (aa)	MW	Crystal structure	Stability
1	Spike	GeneID:43740568	21563	25384	3822	1273	141.2	Yes	Stable
2	Nucleocapsid phosphoprotein	YP_009724397.2	28274	29533	1260	419	45.6	Yes	Unstable
3	Membrane protein	YP_009724393.1	26523	27191	669	222	25.1	No	Stable
4	Envelop	YP_009724392.1	26245	26472	228	75	8.4	Yes	Stable

The virus primarily infects the lung cells that express angiotensin-converting enzyme 2 (ACE2, which is a virus receptor) and is thus responsible for a pneumonia-like disease. ACE2, the main receptor of SARS-CoV-2, is present on various types of cells in the human body, e.g., cells in the heart, kidneys, liver, gastrointestinal tract, nervous system, endocrine system, and some skin sites (Zheng YY. et al., 2020; Pandey et al., 2020). Other receptors which also mediate viral entry include neuropilin-1, AXL, and antibody-FcγR complexes (Zhang et al., 2021b). Neuropilin-1 (NRP1), which is known to bind furin-cleaved substrates, enhances SARS-CoV-2 infectivity. NRP1 is highly expressed in the respiratory and olfactory epithelium, with endothelial and epithelial cells expressing the most. The spike protein’s furin-cleaved S1 fragment binds directly to cell surface NRP1 and disrupting its interaction with a small-molecule inhibitor or monoclonal antibodies inhibited viral infection in cell culture (Cantuti-Castelvetri et al., 2020). The other coronaviruses cause an upper-respiratory-tract infection, whereas SARS-CoV-2 causes a lower-respiratory-tract disease by infecting ciliated cells and type 2 alveolar epithelial cells in the lungs (Astuti and Ysrafil, 2020). SARS-CoV-2 has been detected in the upper respiratory tract too, and these data indicate that the nasopharynx is a site of viral replication. The Spike protein of SARS-CoV and that of SARS-CoV-2 are 76.5% similar, and the binding between the Spike of SARS-CoV-2 and ACE2 is stronger than the binding between SARS-CoV Spike and ACE2 (Zhang et al., 2020).

## 2 The Replication Cycle

Enveloped viruses either enter the cell through direct fusion with the cell membrane and inject their genome or use the alternative mechanism, which involves endocytic machinery. When viruses enter the cell by either of these mechanisms, an additional step of activation is required. For SARS-CoV-2, in the case of direct fusion, the activation is implemented by proteolytic cleavage of Spike, whereas in the endosomal pathway, acidic pH in the endosome is required for successful virus entry. Acidic pH helps the viral and endosomal membranes to fuse and activate proteases. Therefore, the entry of the virus through direct fusion is independent of cellular pH as opposed to the endosomal entry (Yuki et al., 2020).

SARS-CoV-2 is an RNA virus that carries the enzymes required for its replication, because host cells that do not contain enzymes for replicating and translating the RNA genome. Out of the 16 nonstructural proteins that the SARS-CoV-2 genome codes for, two proteins are viral proteases that are responsible for the cleavage of the polyprotein synthesized via translation of the viral genomic RNA. The virus then translates the nonstructural proteins that are required for the replication of the viral genome. After the synthesis of structural proteins, the virion assembly begins. RNA gets packaged inside the nucleocapsid, and the virions are released from the cell, which are now ready to infect other cells (Poduri et al., 2020).

**FIGURE 1:**
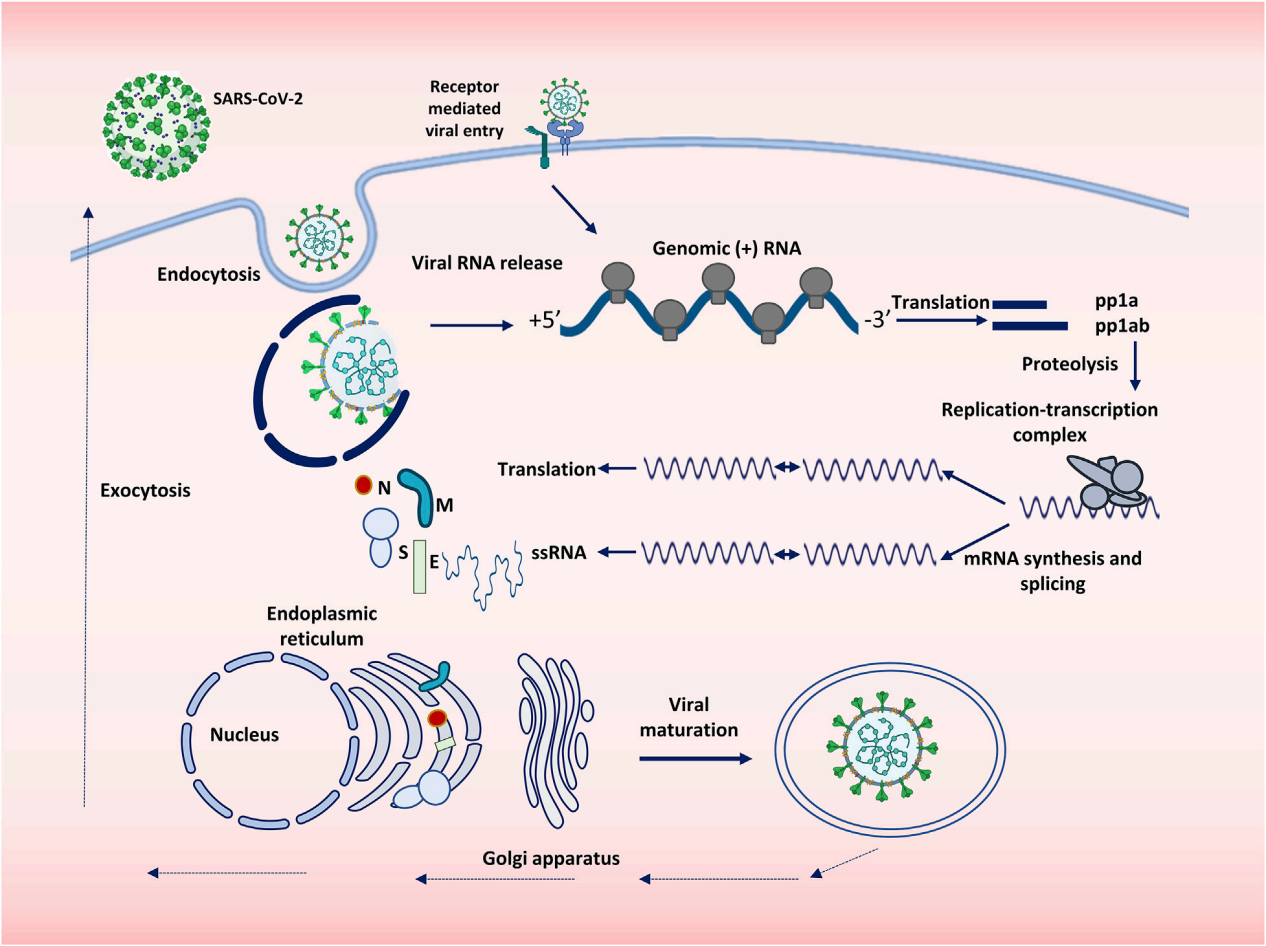
Replication cycle of SARS-CoV-2. SARS-CoV-2 enters the cell either by endocytosis or by receptor mediated membrane fusion. The genome of the virus is released and is subjected to replication and translation. Viral proteins are synthesized, and single stranded RNA is produced, both of which are assembled with the help of Endoplasmic reticulum and Golgi apparatus. The virions thus assembled are then transported outside the body by exocytosis.

SARS-CoV-2 spike is a homotrimeric glycoprotein of 150 kDa. The receptor binding domain (RBD) of spike binds to its receptor called human ACE2 and facilitates the entry of the virus into the host cell (Hoffmann et al., 2020). The virus is activated by the cleavage of the spike glycoprotein into two subunits—S1 and S2—by host cellular proteases, particularly TMPRSS2 (or by lysosomal cathepsins if the virus enters via the endosomal pathway) (Shang et al., 2020a; Hörnich et al., 2021). The first subunit determines cellular tropism, whereas the second one mediates the fusion of a host cell with the virus membrane. After the fusion, the virus enters the cell. Once the virus is inside the cell, it releases its genome into the cytoplasm, where the replication of the genome and its translation into the structural and nonstructural proteins take place (Shang et al., 2020b). SARS-CoV-2 assembly begins with the coating of genomic RNA by the nucleocapsid protein, forming nucleocapsid structures that bud into the endoplasmic reticulum-Golgi intermediate compartment, thereby giving rise to a lipid bilayer containing the viral spike, membrane, and envelope proteins resulting in the formation of the mature virion (Jackson et al., 2021b). The latter then leaves the cell through exocytosis as shown in Figure 1 (Mendonça et al., 2021; Yadav et al., 2021).

The virus downregulates ACE2, as a result of which, the renin angiotensin aldosterone system is dysregulated. This problem leads to tissue injury, vasoconstriction, and increased vascular permeability. Endothelial cells are damaged because of the virus and undergo apoptosis, which results in decreased fibrinolysis and elevated thrombin production. SARS-CoV-2 excessively activates innate immunity and inhibits interferon (IFN) signaling. Consequently, the host develops cytokine release syndrome, wherein excessive production of cytokines occurs, and interleukin 1 beta (IL-1β), IL-6, and IL-10 levels increase. Lymphopenia, a typical condition in severe infections, has been detected in 60–70% of the patients (Gupta et al., 2020).

SARS-CoV-2 infection has a wide variety of clinical manifestations. They range from asymptomatic cases to mild and severe symptoms, and even death. Asymptomatic patients play an especially important role in the COVID-19 pandemic because they spread the infection further without showing any symptoms. It has been reported that 40–50% of the patients were asymptomatic (Oran and Topol, 2020; Wang et al., 2020). Five common clinical manifestations include fever, malaise, cough, sputum secretion, and dyspnea. Comparatively less common symptoms are neurological problems, myalgia, sneezing, sore throat, rhinitis, goosebumps, chest pain, diarrhea etc (da Rosa Mesquita et al., 2021). According to data published in 2020, only 5–10% of all infected individuals develop a severe disease, which includes pneumonia and acute respiratory failure (Raghu and Wilson, 2020). However, the rate of development of severe disease varies based on the age as well as gender (Poletti et al., 2021; Herrera-Esposito and de los Campos, 2022).

## 3 Transmission routes

SARS-CoV-2 is a respiratory virus that primarily infects lower respiratory tract along with the upper respiratory tract and gastrointestinal tract (Ludwig and Zarbock, 2020). It was noticed that when human bronchial epithelial cells were infected with SARS-CoV-2 at a biosafety level 3 lab facility, a large number of virions being produced and released by the cells could be observed by scanning electron microscopy (Ehre, 2020). The main route of transmission in air but it can also be transmitted through the respiratory droplets as well (Port et al., 2022). The main route of transmission is air, but the virus can be transmitted through respiratory droplets as well (Port et al., 2022). Aerosols are also believed to be a possible means of transmission, especially in closed environment (Tellier, 2022; Wilson et al., 2022). Some authors reported that SARS-CoV-2 can survive and maintain its infectivity in an aerosol for hours (Van Doremalen et al., 2020).

Nonetheless, the virus has other transmission pathways in addition to the respiratory route. In a study on organoids, it was proved that the virus is capable of infecting enterocytes (gut cells) that abundantly express ACE2 (the virus receptor). Viral RNA was detected in rectal swabs, suggesting that the fecal-oral route is also a possible transmission pathway (Zhou et al., 2020). Individuals infected with SARS-CoV-2 exhibit gastrointestinal symptoms including diarrhea, nausea, vomiting, and abdominal pain. This observation also points to the possibility that SARS-CoV-2 is an enteric virus (Ding and Liang, 2020; Moura et al., 2022). There is a report of persistent virus shedding by children through feces after their nasopharyngeal swabs tested negative for viral RNA (Xu et al., 2020b). Another study revealed virus shedding through feces even after the disappearance of the virus from nasopharyngeal area (Chen C. et al., 2020). It has been found that the mucosal membrane may also be involved in the transmission of SARS-CoV-2. Close contact with the eyes, nose, or mouth, whether direct or indirect, can lead to COVID-19 transmission (Lu et al., 2021).

## 4 Innate immune sensors and recognition of SARS-CoV-2

**FIGURE 2:**
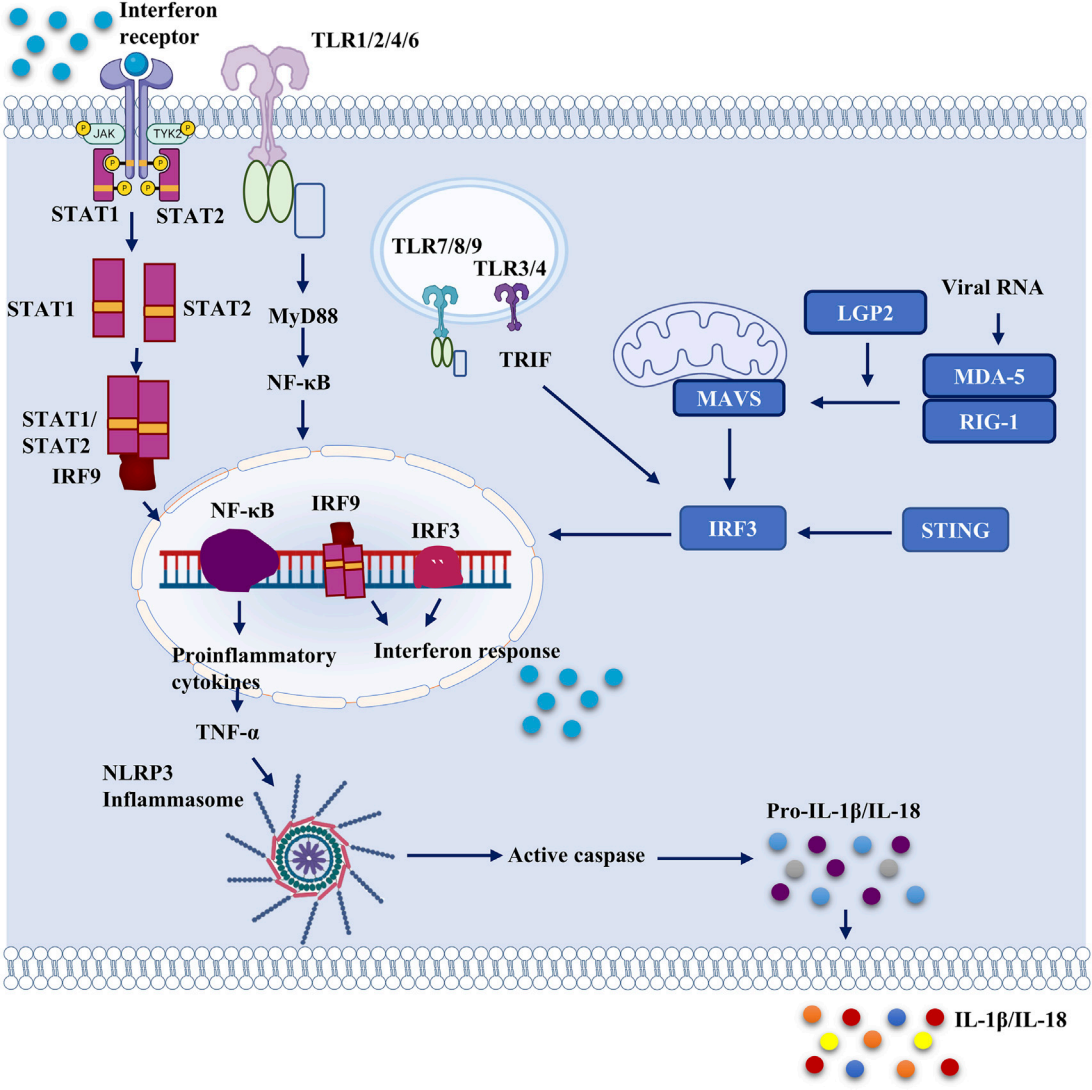
Important pattern recognition receptors and cellular proteins which can serve as potential therapeutic targets against SARS-CoV-2. Different cellular receptors can be activated by viral proteins and viral genome. Structural proteins of SARS-CoV-2 activate membrane bound toll-like receptors and they activate NF-κB by MyD88 activation. Endosomal TLRs are activated by viral structural as well as non-structural proteins, which also activate NF-κB and downstream signaling. Thus, proinflammatory cytokines are released from the cell. Proinflammatory cytokines further activate the NLRP3 inflammasome which leads to secretion of IL-1β and IL-18. NLRP3 inflammasome is activated directly too by viral proteins including ORF3a and non-structural proteins. STING expression is also dysregulated by ORF3a, and 3CLpro. Nucleocapsid and Membrane protein blocks IRF3 phosphorylation and attenuates interferon response. It also interferes with RIG-1. Various SARS-CoV-2 proteins have been reported to interfere with RIG-like receptor signaling.

In order to maintain cell homeostasis and survival, all living organisms require immediate cellular responses to pathogen invasion (Buchmann, 2014). Pattern recognition receptors (PRRs) are germline-encoded cellular receptors that recognize certain patterns of ‘non-self’ and ‘danger’ molecules, referred to as ‘pathogen-associated molecular patterns’ (PAMPs) and ‘danger-associated molecular patterns’ (DAMPs) respectively (DAMPs). PAMPs or DAMPs activate PRRs in mammals, triggering innate immune responses and the production of numerous IFNs and proinflammatory cytokines (Hansen et al., 2011). Several PRRs have been discovered in recent decades, including Toll-like receptors (TLRs), nucleotide-binding oligomerization domain (NOD)-like receptors (NLRs), C-type lectin receptors (CLRs), AIM2-like receptors (ALRs), cyclic GMP-AMP synthase (cGAS) and retinoic acid-inducible gene I (RIG-I)-like receptors (Muñoz-Wolf and Lavelle, 2016). TLRs and RLRs are two of the most important receptors for recognizing RNA virus infection and activating antiviral IFN programs (Chen et al., 2017) (Figure 2).

### 4.1 TLRs and SARS-CoV-2

*Toll* was first identified as an antifungal gene that is key to the *Drosophila* immune system. Subsequent studies further elucidated the fundamental role of TLRs in innate immune sensing in mammals (Lemaitre et al., 1996; Takeda and Akira, 2005). To date, multiple TLRs have been discovered, and their functions have been elucidated. In humans, 10 TLRs (TLR1–TLR10) have been identified, whereas in mice, 12 TLRs (TLR1–TLR9 and TLR11–TLR13) have been discovered, and their functions have been studied (Kawasaki and Kawai, 2014). Each TLR can identify PAMPs that are composed of microbial components such as nucleic acids, lipoproteins, and lipids (Kawai and Akira, 2010). Cell surface TLRs (TLR1/2, 2/6, 4, 5) detect lipids, lipoproteins, and proteins present in microbial membranes as shown in table 3. Endosomal TLRs 3,7,8 and 9 recognize bacterial and viral genomic components; TLR7/TLR8 receptors detect ssRNA, TLR3 detect double-stranded RNA (dsRNA), TLR9 recognizes double stranded DNA in endosomal compartments and recognize viruses that enter through endocytosis (Kawai and Akira, 2007). RLRs and TLRs launch antiviral defense mechanisms as soon as these receptors are engaged by RNA ligands. TLRs bind to adapter proteins such as myeloid differentiation primary response 88 (MyD88; for TLR7 and TLR8) and TIR domain-containing adapter inducing IFN-β (TRIF; for TLR3) to initiate downstream signaling cascades (Vidya et al., 2018).

**TABLE 3:** TLRs, their ligands, cellular localization, and the adaptor molecules.

TLRs	Ligands	Primary localization	Adapter Molecule	References
TLR1	Triacyl lipopolypepties	Cell surface	MyD88	Buwitt-Beckmann et al. (2006); Shinkai et al. (2006)
TLR2	Lipoproteins, zymosan, Pam3CSK4	Cell surface	MyD88	Shinkai et al. (2006); Farhat et al. (2008); Irvine et al. (2013)
TLR3	dsRNA	Intracellular	TRIF	Leonard et al. (2008); Lee et al. (2013)
TLR4	LPS, Viral envelope of glycoproteins	Cell surface and Intracellular	MyD88 and TRIF	Wang et al. (2016); McGettrick and O’Neill, (2010); Cheng et al. (2015)
TLR5	Flagellin	Cell surface	MyD88	Crellin et al. (2005); Huleatt et al. (2008); Huh et al. (2014)
TLR6	Diacyl lipopeptides	Cell surface	MyD88	Pandey et al. (2014); Shinkai et al. (2006)
TLR7	ssRNA	Intracellular	MyD88	Diebold et al. (2004); Maeda and Akira, (2016); Lee et al. (2013)
TLR8	ssRNA	Intracellular	MyD88	Pelka et al. (2016); Shimizu, 2017) (Ishii et al., 2014)
TLR9	Unmethylated CpG- rich DNA, mitochondrial DNA	Intracellular	MyD88	Wagner, (2004); Zhang et al. (2014); Lee et al. (2013)
TLR10	Gp41	Cell surface	MyD88	Henrick et al. (2019); Hasan et al. (2005)

#### 4.1.1 Membrane-Bound TLRs

Studies have revealed that TLR3^−/−^-, TLR4^−/−^-, and TRIF-related adaptor molecule (TRAM)^−/−^- deficient mice are more vulnerable to SARS-CoV infection and show greater transient weight loss when compared to wild-type mice. Mice lacking TRIF (the adaptor protein for TLR3 and TLR4) are more susceptible to SARS-CoV infection (Devarajan and Vaseghi, 2021). Various *in silico* and *in vitro* studies point to the involvement of TLRs in the pathogenesis of COVID-19 (Choudhury and Mukherjee, 2020a; Planès et al., 2021). The injection of INNA-051 (a TLR2/6 agonist) dramatically lowers the amount of viral RNA in the nose and throat in a ferret model (Proud et al., 2021). Another paper indicated that the SARS-CoV-2 envelope protein can upregulate production of pro-inflammatory cytokine through TLR2-dependent signaling (Planès et al., 2021). It is also reported that TLR2 and MyD88 expression affects the severity of the disease. Moreover, TLR2 recognizes the SARS-CoV-2 envelope protein as its ligand and then induces the production of pro-inflammatory cytokines (Sariol and Perlman, 2021; Zheng et al., 2021). Some studies found that the spike protein of SARS-CoV-2 induces a pro-inflammatory response through a TLR-2 mediated pathway (Khan et al., 2021).

TLR4 is expressed in both immune cells and tissue resident cells. The expression of TLR4 and TLR7 was found to be significantly upregulated in mucosal membrane cells in the children with COVID-19; same study reported expression of pro-inflammatory cytokines at both the transcriptional and translational levels (Gankovskaya et al., 2021). TLR4 mediated inflammatory cytokines were found to be upregulated in another study conducted by Sohn et al. (Sohn et al., 2020). It has been proposed that the spike protein of SARS-CoV-2 upregulates TLR4, which ultimately increases the expression of ACE2 on cells and facilitates the entry of SARS-CoV-2. This hypothesis is supported by the finding that ACE2 is expressed by 1–2% of cells in the lungs. Due to ACE2 upregulation mediated by TLR-4, SARS-CoV-2 is able to infect more heart and lung cells (Heads, 2020; Aboudounya et al., 2021; Han et al., 2021; Manik et al., 2022). TAK-242, an anti-inflammatory molecule which inhibits TLR4 mediated signaling, was found to reduce the level of TNF-α and IL-6 in BV-2 mouse microglial cell line (Olajide et al., 2022).

In peripheral blood mononuclear cells, the spike protein is capable of upregulating TLR6 and MMP9 and downregulating SKAP1 and LAG3, which are involved in the control of T-cell function (Zhang et al., 2021a). One of the *in silico* studies suggested that a citrus flavonoid called rutin may be a promising antiviral therapeutic, binding to M-pro, TLR2, TLR6, and TLR7 (Hu et al., 2020). Another *in silico* study indicated that TLR4 has high affinity for the spike protein, and that TLR6 and TLR1 have comparatively lower affinity (Choudhury and Mukherjee, 2020b).

#### 4.1.2 Endosomal TLRs

##### 4.1.2.1 TLR3

TLR3 is an endosomal receptor that recognizes viral dsRNA, thereby inducing the production of type I IFN and inflammatory cytokines (Matsumoto and Seya, 2008). An *in silico* study on the binding of all mRNAs to intracellular TLRs shows that the mRNAs of Nsp10, S2, and E SARS-CoV-2 proteins are potential virus-associated molecular patterns that bind to TLR3, TLR9, and TLR7, respectively, and trigger downstream cascades (Choudhury et al., 2021). Other investigations noticed that SARS-CoV-2 activates TLR3 and TLR7; nevertheless, TLR3 mainly acts through IRF3 activation, whereas TLR7 exerts its action mainly via NF-κB activation (Bortolotti et al., 2021). It has been demonstrated that ORF9b of SARS-CoV-2 inhibits IFN production by targeting the TLR3-TRIF cascade, in addition to RIG-I/MDA-5 MAVS and cGAS-STING signaling pathways (Han et al., 2021). According to a recently published article, polyinosinic:polycytidylic acid (polyI:C; a synthetic TLR3 agonist) protects mice against COVID-19 (Tamir et al., 2022).

##### 4.1.2.2 TLR7

TLR7 recognizes ssRNA and synthetic oligoribonucleotide analogs such as imidazoquinoline, imiquimod, and R-848 (Sun and Reddy, 2013). TLR8 is an endosomal sensor of RNA degradation products in human phagocytes and facilitates the detection of viral and bacterial infections (Moen et al., 2019). In a comparative analysis via an immunoinformatic approach, it was revealed that SARS-CoV-2 has more fragments of ssRNA than SARS-CoV does; therefore, SARS-CoV-2 is capable of hyperactivating the innate immune system by triggering of TLR7/8, resulting in acute lung injury because of the induction of a pro-inflammatory response via the TLR7/8 engagement (Moreno-Eutimio et al., 2020). A loss-of-function mutation in TLR7 increases COVID-19 severity in young males (Moreno-Eutimio et al., 2020). TLR7 agonist imiquimod has been proposed as a possible therapeutic for COVID-19 (Poulas et al., 2020). TLR4 and TLR7 over-expression mediated by SARS-CoV-2 has been detected in adipose tissue-derived mesenchymal stromal cells in response to periodic thermomechanical manipulation (Xu et al., 2021). Due to loss-of-function mutations that suppress the antiviral response to SARS-CoV-2, TLR7 variants constitute a major risk factor for severe COVID-19 in men under the age of 50 (Englmeier and Subburayalu, 2022). In a nested case study, it was found that the loss-of-function mutation of TLR7 is present in 2.1% of the patients with severe COVID-19, but in none of the asymptomatic patients (Fallerini et al., 2021).

Women have higher levels of immune-cell activation than men do, which is linked to TLR7 activation and IFN production (Webb et al., 2019). TLR7 expression is higher in females than in males, and its biallelic expression leads to enhanced immune responses and viral-infection resistance (Spiering and de Vries, 2021). Furthermore, after a viral infection, a lower amount of inflammatory IL-6 is produced in women than in men, and this sign is often associated with a longer lifespan. In addition to this, there are loci on the X chromosome that contain genes implicated in immune-cell modulation (e.g., *FOXP3*) and a transcription factor for regulatory T cells important for viral pathogenesis. The X chromosome affects the immune system by expressing a variety of proteins, including TLR8, CD40L, and CXCR3, all of which can be over-expressed in women and alter the immune system’s response to viral infections and immunizations. That is why women are less susceptible to viral infections than men (Conti and Younes, 2020).

Several TLR7 agonists have been proposed as drugs and vaccine adjuvants against SARS-CoV-2. TLR7 agonists have been conjugated to CoVac501, a self-adjuvanting peptide vaccine. The vaccine is manufactured entirely chemically and incorporates immunodominant peptides identified in the RBD. CoVac501 generates robust and long-lasting titers of protective neutralizing antibodies against numerous RBD mutants, original SARS-CoV-2 strain, and variants in non-human primates (Long et al., 2022).

##### 4.1.2.3 TLR8

An *in silico* study on brain glial cells determined the ability of viral spike epitope peptides M1Lys60 and Ala240Glu300 to bind or interact with human TLR8, with the brain-targeted vascular cell adhesion molecule (VCAM1) protein, with zonula occludens, with glial-cell-specific protein NDRG2, and with apo-S100B. It was also found that a SARS-COV-2 spike peptide forms a heterodimeric complex with human TLR8 (Dasgupta and Bandyopadhyay, 2021). A bioinformatic analysis of the viral genome revealed hundreds of fragments that might activate TLR7/8, suggesting that products of endosomal processing of the virus can potently induce IFN-based and inflammatory responses downstream of these receptors. SARS-CoV-2 associated molecular patterns elicit MyD88-dependent lung inflammation in mice, which is characterized by over-expression of pro-inflammatory and cytotoxic mediators, immune-cell infiltration, and phenotypic maturation of splenic dendritic cells. Thus, TLR7/8 was discovered as a critical cellular sensor of SARS-CoV-2 ssRNAs that takes part in host resistance and in COVID-19 development (Salvi et al., 2021).

In another research article, it is demonstrated that in the absence of pyroptosis, GU-rich ssRNA (GU-rich RNA) derived from SARS-CoV-2, SARS-CoV-1, or HIV-1 launches a TLR8-dependent pro-inflammatory cytokine response in human macrophages, with GU-rich RNA from the SARS-CoV-2 spike gene triggering a pro-inflammatory response. Moreover, it was shown using genetic and pharmacological suppression that the production of mature IL-1β is mediated by a non-classic pathway that is dependent on caspase 1, caspase 8, the NLRP3 inflammasome, potassium ion efflux, and autophagy while being independent of TRIF (TICAM1), vitamin D_3_, and pyroptosis (Campbell et al., 2021).

*TLR8* mRNA has turned out to be more abundant than *TLR7* mRNA, which indicates the presence of monocyte-derived dendritic cells (MDDCs). In *in vitro* assays on MDDCs activated with ssRNA40 (a TLR8 agonist that is a positive-sense ssRNA similar to SARS-CoV-2 RNA), upregulation of sXBP1 and mRNA and protein expression of IL-1β, IL-6, and TNF was noticed. An IRE1 ribonuclease inhibitor (MKC8866) suppressed these responses. Moreover, 1) the presence of sXBP1 in nasopharyngeal swabs and BAAs, 2) sXBP1 induction by ssRNA40 in MDDCs, 3) the effect of IRE1 RNase inhibition on the cytokine induction produced by ssRNA40, and 4) the demonstration of sXBP1 binding to *IL1B*, *IL6*, and *TNF* promoters, all suggest that TLR8-induced XBP1 splicing potentially contributes to the viral sepsis observed in severe cases of COVID-19 (Fernández et al., 2022).

##### 4.1.2.4 TLR9

It has been hypothesized that for COVID-19, TLR9 activation is a subtle but potent factor causing hyperinflammation and thrombotic complications in vulnerable patients. As a result, testing the efficacy of TLR9 agonists is recommended for the fight against SARS-CoV-2 (Oberemok et al., 2020).

Human mitochondrial DNA, which is derived from endosymbiont bacteria and contains unmethylated CpG motifs, is a model of a well-known DAMP that directly activates the inflammatory process via TLR9 upon tissue damage and infection (Zhang et al., 2010). In endothelial cells, SARS-CoV-2 infection affects mitochondrial function and promotes TLR9 signaling. TLR9 launches inflammatory responses that culminate in endothelial-cell dysfunction, which may contribute to the severity of COVID-19 symptoms. Targeting of mitochondrial metabolic pathways may help to devise new COVID-19 treatments (Costa et al., 2022).

CpG 1018, which is a T helper 1 (Th1)-biasing synthetic TLR9 agonist, has been chosen as an adjuvant candidate to improve immunogenicity and attenuate possible vaccine-induced immunopathology. Cytokines generated by splenocytes of mice vaccinated with CpG 1018 and alum show a strong Th1-dominant response. In a dose-ranging trial, rats subjected to single- or double-dose regimens of S-2P coupled with CpG 1018 alone or CpG 1018 plus alum manifested no vaccine-related significant adverse effects. These findings support the development of a COVID-19 vaccine candidate based on CHO-derived S-2P formulated with CpG 1018 and alum (Kuo et al., 2020).

In another study, several combinations of PRR agonists and aluminum hydroxide (AH) were compared, and it was discovered that combining CpG oligodeoxynucleotides (TLR9 agonists) with AH and RBD significantly improves immune responses to RBD in young mice on a prime-boost immunization schedule. In aged mice, the AH:CpG-adjuvanted RBD vaccination evoked a strong anti-RBD immune response, with an extra boost dose generating an anti-RBD antibody response comparable to that in young adult mice and conferring complete protection against a live-SARS-CoV-2 challenge. In addition, it was observed that the AH:CpG adjuvant formulation has unique immunological capabilities yielding human-leukocyte activation *in vitro*. Overall, the thorough adjuvant comparison analysis showed that co-adjuvanting AH:CpG can overcome both RBD immunogenicity and immunosenescence (Nanishi et al., 2021).

Haabeth et al. (2021) looked into the development of a new mRNA vaccine that involves charge-altering releasable transporters as a delivery mechanism. It was proposed that vaccination immunogenicity could be customized by including co-formulated adjuvants such as oligodeoxynucleotides with CpG motifs (CpG-ODN) in these intrinsically nonimmunogenic vehicles. Mice given the mRNA-charge-altering-releasable transporter vaccine acquired therapeutically relevant levels of RBD-specific neutralizing antibodies in the blood and bronchial secretions. Furthermore, the vaccination induced RBD-specific Th1 T-cell responses, including CD4^+^ and CD8^+^ T-cell memory, that were strong and long-lasting (Haabeth et al., 2021).

Various drugs which target toll-like receptors are being evaluated in clinical trials. Some of them have been listed in table 4.

**TABLE 4:** Clinical trials of the drug targeting immune mediators against SARS-CoV-2.

Name	Molecular target or pathway involved	Identifier number	Last updated	Status	Phase
Melatonin	NLRP3 Inflammasome	NCT04353128	23 November 2021	Completed	Phase 3
		NCT04474483	11 June 2021	Recruiting	Phase 2
		NCT04784754	11 June 2021	Recruiting	Phase 2
		NCT04409522	1 June 2020	Recruiting	NA
		NCT04470297	24 July 2020	Not recruiting yet	Phase 2
Curcumin		NCT05150782	11 January 2022	Not recruiting yet	NA
		NCT05130671	28 January 2022	Completed	NA
		NCT04802382	29 June 2021	Recruiting	Phase 3
		NCT04382040	31 August 2021	Completed	Phase 2
		NCT05037162	8 September 2021	Not recruiting yet	Phase 2
		NCT04844658	1 December 2021	Completed	NA
Indomethacin		NCT05007522	26 November 2021	Not recruiting yet	Phase 3
		NCT04344457	1 July 2020	Recruiting	Phase 1; Phase 2
Pioglitazone		NCT04500639	3 May 2021	Recruiting	NA
Metformin		NCT04604678	7 January 2021	Not recruiting yet	Phase 2
		NCT04625985	8 April 2021	Completed	Phase 2
		NCT04510194	26 January 2022	Recruiting	Phase 3
Colchicine		NCT04322565	10 July 2020	Recruiting	Phase 2
		NCT05118737	3 December 2021	Recruiting	Early phase 1
Statins	ox-LDL-mediated NLRP3 inflammasome activation	NCT05238402	14 February 2022	Completed	NA
		NCT04472611	2 March 2021	Recruiting	Phase 3
		NCT04904536	7 October 2021	Not yet recruiting	Phase 3
Resveratrol	TLR4	NCT04799743	16 July 2021	Recruiting	NA
		NCT04666753	14 December 2020	Completed	NA
M5049	TLR7/8 antagonist	NCT04448756	8 September 2021	Completed	Phase 2
Poly I:C	TLR3 agonist	NCT04672291	18 August 2021	Recruiting	Phase 1
PUL-042	TLR2/6/9 agonist	NCT04312997	16 July 2021	Completed	Phase 2
		NCT04313023	2 September 2021	Completed	Phase 2
Hydroxychloroquine sulfate	TLR7/9 inhibitor	NCT04340349	29 June 2021	Enrolling by invitation	Early phase 1
Baricitinib	Janus kinase inhibitor	NCT05082714	30 November 2021	Recruiting	NA
		NCT04390464	18 May 2020	Recruiting	Phase 4
		NCT04421027	19 July 2021	Completed	Phase 3
		NCT04970719	27 August 2021	Recruiting	Phase 3
		NCT05056558	28 September 2021	Not yet recruiting	Phase 3
		NCT04693026	5 January 2021	Recruiting	Phase 3
		NCT05074420	19 January 2022	Recruiting	Phase 3
		NCT04832880	6 April 2021	Not yet recruiting	Phase 3
		NCT04390464	18 May 2020	Recruiting	Phase 4
Ruxolitinib		NCT04362137	11 October 2021	Completed	Phase 3
		NCT04334044	7 July 2021	Completed	Phase 1; Phase 2
		NCT04338958	13 August 2021	Completed	Phase 2
		NCT04348071	8 March 2021	Withdrawn	Phase 2; Phase 3
		NCT04414098	4 June 2020	Not yet recruiting	Phase 2
		NCT04359290	24 August 2021	Completed	Phase 2
		NCT04348695	17 April 2020	Recruiting	Phase 2
		NCT04581954	2 September 2021	Recruiting	Phase 1; Phase 2
Secukinumab	IL-17A antagonist	NCT04403243	27 May 2020	Recruiting	Phase 2

### 4.2 RIG-Like Receptors (RLRs) and SARS-CoV-2

RLRs, unlike TLRs, are crucial cytoplasmic virus sensors that detect intracellular non-self RNAs with different secondary-structure patterns or metabolic changes (Lässig and Hopfner, 2017). RLRs are RNA helicases that include the Asp-Glu-Ala-Asp (DEAD) box, and this family includes three members: RIG-I, melanoma differentiation-associated gene 5 (MDA5), and laboratory of genetics and physiology 2 (LGP2). All RLRs have an RNA helicase domain with a C-terminal domain, which is responsible for RNA binding. Although RIG-I and MDA5 both contain tandem caspase activation and recruitment domains (CARDs) for downstream signal transduction, LGP2 does not have a CARD and therefore acts as a modulator of RIG-I and MDA5 (Yoo et al., 2014). RIG-I and MDA5 have similar as well as separate mechanisms for recognizing non-self RNAs. As mammalian cells lack an RdRp, dsRNA is a characteristic non-self RNA that is normally absent in uninfected cells. An artificial dsRNA called polyI:C can activate both RIG-I and MDA5 (Schlee and Hartmann, 2016). Of note, RIG-I and MDA5 activation by dsRNA is regulated differently depending on the length of the dsRNA. The shortest dsRNA found to activate RIG-I is 13 bp, which is the same as the shortest length required for the assembly of a 2-RIG-I/dsRNA dimer (Brisse and Ly, 2019). Recent research uncovered several biochemical properties of RLR-activating RNA species, including 1) 5′-triphosphate in RNAs having a secondary structure, 2) 5′-diphosphate and the absence of a cap, and 3) the presence of an unmethylated 5′-end nucleotide at the 2′-O position; such RNAs are typically generated during the replication of RNA viruses, including coronaviruses (Rehwinkel and Gack, 2020).

RLRs undergo conformational changes driven by ATPase/helicase activity, from an inactive “closed” state to an active “open” state. The CARD-CARD interaction allows activated RLRs to release CARDs, which then come into contact with a signaling adaptor molecule called mitochondria antiviral signaling protein (MAVS) (Leung and Amarasinghe, 2012). Signaling adapters MyD88, TRIF, and MAVS then recruit several ubiquitin ligases, such as TNF receptor-associated factor (TRAF) 3 and TRAF6, which associate with antiviral kinases like TANK-binding kinase 1 (TBK1), IκB kinase (IKK), and the IKK complex, to coordinate downstream signaling pathways. As a result of the activation of transcription factors IRF3 and IRF7 as well as NF-κB, type I IFN and pro-inflammatory cytokines are produced, allowing host antiviral IFN systems to function freely (Konno et al., 2009; Kawasaki and Kawai, 2014). It was found *in vitro* experiments that IFNs inhibit the replication cycle of SARS-CoV-2 in a dose-dependent manner (Busnadiego et al., 2020; Vanderheiden et al., 2020).

Yin et al. (2021) reported the involvement of MDA5 and LGP2 (members of the RLR family) in the induction of an IFN response as a consequence of SARS-CoV-2 infection. They also identified IRF3, IRF7, and NF-κB as the key transcription factors responsible for the regulation of IFN production in lung epithelial cells during SARS-CoV-2 infection (Yin et al., 2021). Another group demonstrated that the MDA5 protein mediates IFN production in lung cells as a result of COVID-19; however, the enhanced IFN response could not control the viral replication in the lung cells (Rebendenne et al., 2021). Another research group proposed that the RNA sensing through MDA5 is the key driver of inflammation in lung cells. They also suggested that the inhibition of innate immune sensing of SARS-CoV-2 may alleviate the inflammation caused by COVID-19 (Thorne et al., 2021). Different research groups have reported that different proteins of SARS-CoV-2 interfere with RIG1 and MDA5 or with either RIG1 or MDA5 signaling. One group reported that PL-pro antagonizes ISG15-dependent activation of MDA5 by de-ISGylation. MDA5 oligomerization is triggered by ISGylation of CARDs, and this process triggers innate immunity against a variety of viruses such as coronaviruses, flaviviruses, and picornaviruses. Accordingly, this mechanism helps the virus to evade the immune response (Liu et al., 2021b). By contrast, other studies reported that the M protein of SARS-CoV-2 suppresses type I and type III IFN responses by preventing the phosphorylation and activation of IRF3 through interaction with RIG-I, MAVS, and TBK1 (Zheng Y. et al., 2020). The nucleocapsid protein inhibits IFN secretion by blocking IRF3 phosphorylation and by downregulating Tyk2 and STAT2 (Kumar et al., 2021).

Recently, it was observed that MDA5 is over-expressed in asymptomatic and mildly symptomatic patients. Type I IFN was also found to be significantly upregulated in nasopharyngeal cells of the asymptomatic patients. This result points to a protective role of MDA5 involving upregulation of type I IFN to protect the patient from possible severe tissue damage caused by SARS-CoV-2 (Mohanty et al., 2021). The nucleocapsid protein of SARS-CoV-2 has been reported to inhibit the interaction between tripartite motif protein 25 (TRIM25) and RIG-I, as well as to suppress nuclear translocation of IFN-regulatory factor 3, thereby attenuating IFN production (Oh and Shin, 2021). Other components of SARS-CoV-2 have also been found to inhibit the induction of an IFN response. ORF3b, ORF6, ORF7a, ORF8, and Nsp14 in addition to the Nucleocapsid protein of SARS-CoV-2 are potent inhibitors of an IFN response (Li JY. et al., 2020; Cao et al., 2021; Hsu et al., 2021; Redondo et al., 2021).

### 4.3 NLRs and SARS-CoV-2

Inflammasomes are multiprotein complexes composed of members of the NLR family and of the pyrin and HIN domain (PYHIN) family. Inflammasomes’ biochemical purpose is to trigger caspase 1, which induces the maturation of IL-1β and IL-18 as well as the activation of pyroptosis, a type of cell death. The roles of the NLRP3 inflammasome in human immunity and pathologies have been proven extensively. The NLRP3 inflammasome is a crucial mediator of host immune responses because it activates caspase 1 and IL-1β and/or IL-18 (He et al., 2016).

The NLRP3 inflammasome, unlike other inflammasomes, can be activated by a variety of stimuli. The NLRP3 inflammasome is thought to sense cellular homeostasis rather than directly recognize a common motif present in its inducers; many physiological signals, e.g., K^+^ efflux, Ca^2+^ signaling, mitochondrial malfunction, and lysosomal rupture, have been hypothesized to drive its activation (He et al., 2016). Infectious microorganisms such as the Sendai virus, influenza virus, adenovirus, fungi *Saccharomyces cerevisiae* and *Candida albicans*, and bacteria (such as *Staphylococcus aureus*, *Listeria monocytogenes*, and *Shigella flexneri*) are also able to activate the NLRP3 inflammasome. In some cases, specific microbial components or products that activate the NLRP3 inflammasome have been identified, such as bacterial and viral RNA, malaria-causing parasite hemozoin crystals, and various bacterial pore-forming toxins (Chengcheng et al., 2010; Zhao N. et al., 2021). The ORF3a-encoded SARS-CoV-2 viroporin—a type of tiny hydrophobic protein encoded by viruses that oligomerizes in host cell membranes resulting in the formation of hydrophilic pores—activates the NLRP3 inflammasome (Nieva et al., 2012). ORF3a activates the inflammasome via either an ASC-dependent or ASC-independent mechanism by triggering IL-1β expression. ORF3a-mediated inflammasome activation necessitates K^+^ efflux and NEK7-NLRP3 oligomerization (Xu et al., 2022). MCC950, a small-molecule inhibitor of NLRP3’s ATPase activity, lowers the production of IL-18 in response to these viral proteins (Rousseau et al., 2021). It was observed in another study that MCC950 can block ORF3a-mediated inflammasome activation. ORF3a key residues needed for the virus release and inflammasome activation are conserved among SARS-CoV-2 isolates across continents, suggesting that ORF3a and NLRP3 are prime targets for a therapeutic intervention (Xu et al., 2020a).

Additionally, NLRP3 leads to the release of several biologically active DAMPs. This action sets off a chain of events that result in amplification of the innate immune system’s reaction and trigger the complement cascade (ComC). In addition to DAMPs, mannan-binding lectin (MBL), which binds to SARS-CoV-2 proteins, has been shown to directly launch the ComC. Notably, activation of the ComC via the MBL-MASP-2 protease complex also triggers the coagulation cascade (CoaC), and the launch of the latter is associated with a worse prognosis in patients infected with SARS-CoV-2. This observation explains why ComC and CoaC inhibition are individually being investigated as a therapeutic option (Ratajczak and Kucia, 2020). It has been demonstrated that NLRP3 is activated by SARS-CoV-2 at an early stage of the infection and induces cytokine release syndrome. These data suggest that NLRP3 can serve as an effective target for reducing organ damage (Marchetti et al., 2021).

Yalcinkaya et al. (2021) reported that the E protein prevents inflammasome priming and NLRP3 inflammasome activation in cultured macrophages. Similarly, in animals transfected with the E protein and treated with polyI:C mimicking the effects of viral RNA, the E protein reduces pro-IL-1β expression, IL-1β and IL-18 levels in bronchoalveolar lavage fluid, and macrophage infiltration of the lungs in an NLRP3-dependent manner. Macrophages were treated with both LPS and polyI:C to simulate the consequences of a more advanced infection. Under these conditions, the E protein boosted the activation of the NLRP3 inflammasome in both murine and human macrophages. As a consequence, the SARS-CoV-2 E protein may reduce the host NLRP3 inflammasome response to viral RNA at early stages of the infection while possibly enhancing NLRP3 inflammasome responses later on. The mentioned study suggests that targeting the SARS-CoV-2 E protein, particularly in the early stages of the infection, may be a unique way to treat COVID-19 (Yalcinkaya et al., 2021).

Two non-structural proteins, Nsp1 and Nsp13, suppress caspase 1-mediated IL-1β activation, according to a screening of a complementary DNA (cDNA) library containing 28 SARS-CoV-2 ORFs. Caspase 1 inhibition was attributed to Nsp1 amino acid residues involved in the silencing of host translation and to Nsp13 domains responsible for helicase activity. Both Nsp1 and Nsp13 attenuated NLRP3 inflammasome-induced caspase 1 activity and IL-1β production in THP-1 cells. These data suggest that SARS-CoV-2 Nsp1 and Nsp13 are effective NLRP3 inflammasome antagonists (Kim et al., 2021b).

A recent mutational analysis of SARS-CoV-2 implies that Nsp6 is an important indicator of viral pathogenicity. Inflammasome-related NOD-like receptor signaling is activated in SARS-CoV-2-infected lung epithelial cells and COVID-19 patients’ lung tissues, according to transcriptome analysis. The induction of inflammasomes/pyroptosis in patients with severe COVID-19 was validated by means of serological markers. Nsp6 over-expression resulted in NLRP3/ASC-dependent caspase 1 activation, maturation of IL-1β and IL-18 or either IL-1β or IL-18, and pyroptosis of lung epithelial cells. Nsp6 inhibited autophagic flux by impairing lysosome acidification, which was reversed by 1,25-dihydroxyvitamin D_3_, metformin, or polydatin, which prevented Nsp6-induced pyroptosis. Nsp6 interacts directly with ATP6AP1 (a proton pump component of vacuolar ATPase) and reduces its cleavage-mediated activation. Reduced binding to ATP6AP1 and a diminished ability to impede lysosome acidification for promoting pyroptosis were revealed in the L37F Nsp6 variant, which was linked with asymptomatic COVID-19. Infection of cultured lung epithelial cells with live SARS-CoV-2 caused autophagic-flux stagnation, inflammasome activation, and pyroptosis in a consistent manner. Overall, the mentioned research suggests that SARS-CoV-2 Nsp6 can cause inflammatory cell death of lung epithelial cells, and this effect could be exploited therapeutically through pharmacological correction of autophagic flux (Sun et al., 2022).

In a confocal immunofluorescence analysis, the SARS-CoV-2 nucleocapsid was found to co-localize with neurons, astrocytes, oligodendrocytes, and microglia. In cerebral cortical tissues, the viral nucleocapsid was discovered alongside ACE2 (cell entry receptor) and the NLRP3 inflammasome (Cama et al., 2021). In another study, it was reported that the N protein interacts directly with the NLRP3 protein and facilitates NLRP3 inflammasome assembly by promoting NLRP3 binding to ASC (Pan et al., 2021).

The spike protein of SARS-CoV-2 has also been found to activate the NLRP3 inflammasome (Kucia et al., 2020). In another research article, it is reported that in cultured murine alveolar macrophages, ultramicronized palmitoylethanolamide inhibits NLRP3 inflammasome expression and the pro-inflammatory response activated by the SARS-CoV-2 Spike protein (Del Re et al., 2021). It was also observed that the infected cells undergo pyroptosis mediated by inflammasome activation involving NLRP3 and AIM2. Moreover, tissue-resident macrophages show inflammasome activation, but this phenomenon is not observed in infected epithelial cells (Junqueira et al., 2021). The SARS-CoV-2 spike protein triggers inflammasome activation and an IL-1β release in macrophages obtained from COVID-19 patients but not in macrophages from healthy SARS-CoV-2-naive controls. *Ex vivo*, chemical NLRP3 suppression inhibits spike protein-induced IL-1β production (Theobald et al., 2021). Another study indicated that ORF3a is required for the assembly of the NLRP3 inflammasome and for the secretion of mature IL-18. Albeit to a lesser extent, Nsp2 and ORF3b (among 26 tested viral proteins) enhanced NLPR3 inflammasome activity and the production of mature IL-18. In human-*ACE2* transgenic mice, specific suppression of the NLRP3 inflammasome by MCC950 reduces excessive lung inflammation and hence COVID-19-like pathology (Zeng et al., 2022).

In infected human monocytes, SARS-CoV-2 infection activates the NLRP3 inflammasome and inhibits caspase 1, GSDMD cleavage, and pyroptosis. *in vitro* and *in vivo* experiments, the SARS-CoV-2 nucleocapsid protein interacts with GSDMD in cells and suppresses GSDMD cleavage. The Nucleocapsid protein attaches to the GSDMD linker region, thus preventing caspase 1 from processing this protein (Ma et al., 2021). In human microglial cells, NLRP3 could be primed and activated by the Spike protein of SARS-CoV-2 in an NF-κB-dependent as well as ACE2-dependent manner (Albornoz et al., 2022). Thus, it becomes very clear that NLRP3 is an attractive target to be probed against SARS-CoV-2. Drugs which are under clinical trials have been listed in table 4.

### 4.4 CLRs and SARS-CoV-2

CLRs, which belong to the superfamily of membrane-bound phagocytic PRRs, recognize carbohydrates on the surface of pathogens in a Ca^2+^-dependent manner and hence are termed “C-type lectin receptors.” CLRs play a substantial part in the regulation of adaptive immune responses by managing the induction of signaling pathways. CLRs can regulate adaptive immunity on multiple levels by initiating signaling on their own, through interaction with other PRRs, or by triggering carbohydrate-specific signaling cascades (Geijtenbeek and Gringhuis, 2009; Li and Wu, 2021). They can either modulate TLR signaling or directly induce gene expression. CLRs can be soluble or transmembrane receptors (Kerrigan and Brown, 2010), and are expressed by antigen-presenting cells such as dendritic cells, macrophages, monocytes, and Langerhans cells (Bermejo-Jambrina et al., 2018). The participation of CLRs in virus recognition is complicated. They can detect and respond efficiently to a wide spectrum of viruses and thus can shape antiviral immune responses (Monteiro and Lepenies, 2017).

Several C-type lectins (DC-SIGN, L-SIGN, LSECtin, ASGR1, and CLEC10A) serve as glycan-dependent binding partners of the spike protein of SARS-CoV-2. These substances interact with spike through the region around the RBD (Lu et al., 2021). Recently, it was demonstrated that attachment to such receptors as CLRs, DC-SIGN, L-SIGN, and sialic-acid-binding immunoglobulin-like lectin 1 (SIGLEC1) enhances ACE2-mediated infection and modulates the neutralizing activity of distinct types of spike-specific antibodies (Rahimi, 2020; Amraei et al., 2021; Lempp et al., 2021). Another study revealed that DC-SIGN, L-SIGN, langerin, and macrophage galactose-type C-type lectin can interact with the spike protein of SARS-CoV-2, and that DC/L-SIGN facilitates the virus entry into the cell through ACE2. Nonetheless, by means of a glycol-mimetic compound designed against DC-SIGN, *trans*-infection between different cell types can be inhibited (Thépaut et al., 2021). Human liver sinusoidal endothelial cells and lymph node lymphatic endothelial cells express L-SIGN abundantly, whereas blood endothelial cells do not. SARS-CoV-2 proteins have been found within liver sinusoidal endothelial cells in liver autopsy samples from COVID-19 patients by high-resolution confocal microscopy. In comparison with control cells, both a pseudo-typed virus wrapped in the SARS-CoV-2 spike protein and the genuine SARS-CoV-2 virus infect L-SIGN-expressing cells. Furthermore, inhibition of L-SIGN function reduces SARS-CoV-2 infection. These findings suggest that L-SIGN is a receptor mediating SARS-CoV-2 infection (Kondo et al., 2021).

### 4.5 cGAS-STING Pathway

Cyclic GMP-AMP (cGAMP) synthase (cGAS) cytosolic DNA sensor triggers innate immune responses by producing cGAMP, the second messenger, which activates the interferon gene adaptor stimulator (STING). The cGAS-STING system mediates protective immune protection against a wide range of DNA-containing pathogens (Chen et al., 2016). Increasing data suggest that RNA viruses can elicit cGAS-STING responses and be blocked by cGAS-STING activation in general (Ishikawa and Barber, 2008). Bats are believed to be a natural host for many coronaviruses, and all bats studied so far have STING molecules that are faulty in inducing type-I IFN and have limited antiviral efficacy (Xie et al., 2018). The duration of coronavirus infection in bats has been associated with faulty STING activation. During cross-species transmission, SARS-CoV-2 appears to have retained or regained its ability to block the STING function. SARS-CoV-2 inhibits cGAS-STING activity with at least two viral proteins. SARS-CoV-2 3CLpro and ORF3a can antagonize various vertebrate STING functions, including those of humans, mice, and chickens, which is consistent with the fact that SARS-CoV-2 can infect a wide spectrum of species (Rui et al., 2021).

Through transcriptome analysis, SARS-CoV-2 was found to be responsible for the activation of the cGAS-STING pathway and NF-κB activation. This suggests that inhibition of STING can ameliorate the upregulated cytokine production (Neufeldt et al., 2022). Furthermore, within cells expressing ACE2 and the SARS-CoV-2 spike protein, viral infection can cause the development of syncytia, resulting in the creation of micronuclei. Micronucleus translocation cGAS, as well as overexpression of their downstream target genes, revealed that these micronuclei had a high level of activation of both the DNA damage response and cGAS-STING signaling. (Ren et al., 2021). The STING small-molecule agonist has been tested against SARS-Cov-2 infection in vitro and preclinical studies and has been found to stimulate interferon signaling and antiviral state against the virus. Intranasal delivery of diABZI-4, small molecule agonist of STING, was found to activate STING and proinflammatory cytokines (Humphries et al., 2021). In another study, diABZI significantly inhibited SARS-CoV-2 infection by inducing interferon signaling both *in vitro* and *in vivo* experiments (Li et al., 2021). These studies suggest the importance of cGAS**-**STING pathway in interferon signaling in response to SARS-CoV-2 thus pointing out to the need of conducting clinical trials for further validation of using STING agonist as a clinical drug.

## 5 Inflammatory Cytokines and the Cytokine storm in COVID-19

Immunopathological manifestations of COVID-19 include lymphopenia, dysregulated macrophages and monocytes, neutrophilia, antibody-dependent enhancement, a reduced or delayed IFN response, and a cytokine storm (Yang et al., 2021). The latter is defined as an immunological state marked by rapid proliferation and over-activation of T cells, macrophages, and natural killer cells as well as over-production of more than 150 inflammatory cytokines and chemical mediators released by the immune cells and nonimmune cells (Rabaan et al., 2021) The term “cytokine storm” was first used in 1933, in reference to acute graft-versus-host disease in the context of hematopoietic stem cell transplantation (Kim et al., 2021a). After the successful recognition of a virus by the immune system in the human body, the virus is presented to natural killer cells and CD8^+^ cells in the form of viral antigens by antigen-presenting cells, which subsequently activate immune responses (Liskova et al., 2021). Cytokine and chemokine responses are a part of innate as well as adaptive immune responses because they play a crucial role in communication between different cells (Lacy, 2015). It has been reported that in a large proportion of cases, the severity of COVID-19 is explained mainly by hyper-inflammation and the cytokine storm (Kim et al., 2021a). The latter also contributes to deterioration of the health condition in a large number of COVID-19 patients by promoting acute respiratory distress syndrome, multiple organ dysfunction, and coagulation deficits (Chen et al., 2021).

Secretion of multiple cytokines, also known as cytokine release syndrome, is linked with the emergence of clinical symptoms. For example, IFN-γ can cause fever, chills, headache, dizziness, and fatigue. TNF can induce not only flu-like symptoms such as fever, general malaise, and fatigue, but also can cause vascular leakage, cardiomyopathy, lung injury, and acute-phase protein synthesis. IL-6, a key target in adoptive-cell therapy-induced cytokine release syndrome, can cause vascular leakage, complement activation, and triggering of the coagulation cascade, leading to severe symptoms of cytokine release syndrome such as diffuse intravascular coagulation and multiple organ failure (Shimabukuro-Vornhagen et al., 2018). It is worth noting that IL-6 may cause cardiomyopathy by promoting cardiac dysfunction, which is common in patients with cytokine release syndrome (Sun et al., 2020). It is reported that patients with COVID-19 symptoms have higher levels of cytokines and inflammatory markers such as IL-6, IL-8, IL-1β, IL-10, IL-12, and IFN-γ (Costela-Ruiz et al., 2020). At present, there is a strong link between higher levels of pro-inflammatory cytokines—as potential markers of a cytokine storm—and the severity of COVID-19 as well as poor outcomes in hospitalized patients (Del Valle et al., 2020). Elevated IL-6 concentration is recognized as one of the most important markers of unfavorable outcomes and serves as an indicator of disease progression (Santa Cruz et al., 2021).

A stressed or infected cell activates a vast number of white blood cells, including B cells, T cells, natural killer cells, macrophages, dendritic cells, and monocytes, through receptor-ligand interactions. Inflammatory cytokines are released, which stimulate additional white blood cells in a positive feedback loop. After a primary infection, a cytokine storm begins locally and spreads throughout the body via systemic circulation. Calour (heat), dolour (pain), rubor (redness), tumor (swelling or edema), and loss of function (functio laesa) are all classic indicators of inflammation (Fara et al., 2020a). Initially, the localized response involves defensive mechanisms such as increased blood flow, facilitation of leucocyte extravasation and supply of plasma proteins to the site of injury, an increase in body temperature (advantageous in the case of bacterial infections), and pain triggering (Askenase and Sansing, 2016). Soon after the pathogenic trigger, repair mechanisms are initiated. There are two likely outcomes of these events: 1) organ function is progressively restored and 2) healing occurs via fibrosis, which can lead to chronic organ dysfunction (Fara et al., 2020b).

Several drugs that act via the blockade of the upregulated cytokines are currently in clinical trials against COVID-19. Some of them are listed in Table 5.

**TABLE 5:** Clinical trials of the drugs targeting cytokines different signaling molecules involved in the immune response against SARS-CoV-2.

Name	Molecular target or pathway involved	Identifier number	Last updated	Status	Phase
Tocilizumab	IL-6 inhibitor	NCT04730323	29 January 2021	Completed	Phase 4
		NCT04445272	2 June 2021	Completed	Phase 2
		NCT04479358	2 December 2021	Recruiting	Phase 2
		NCT04924829	14 June 2021	Recruiting	NA
		NCT04412772	17 November 2020	Recruiting	Phase 3
		NCT04332094	6 May 2021	Recruiting	Phase 2
Sarilumab		NCT04386239	20 July 2021	Recruiting	Early phase 1
		NCT04357808	11 February 2021	Completed	Phase 2
		NCT04315298	23 September 2021	Completed	Phase 2; Phase 3
		NCT04661527	16 December 2020	Recruiting	Phase2
		NCT04359901	8 March 2021	Active, not recruiting	Phase 2
		NCT04357860	7 July 2021	Completed	Phase 2
		NCT04327388	13 May 2021	Completed	Phase 3
		NCT04322773	9 October 2020	Terminated	Phase 2
Clazakizumab		NCT04348500	2 December 2021	Completed	Phase 2
		NCT04381052	23 September 2021	Completed	Phase 2
		NCT04494724	31 July 2020	Recruiting	Phase 2
		NCT04363502	14 June 2021	Recruiting	Phase 2
		NCT04343989	15 February 2022	Completed	Phase 2
Anakinra	IL-1 Receptor antagonist	NCT04443881	1 June 2021	Completed	Phase 2; Phase 3
		NCT04603742	25 January 2022	Withdrawn	Phase 2
		NCT04594356	30 August 2021	Active, not recruiting	NA
		NCT04366232	16 December 2020	Terminated	Phase 2
		NCT04412291	18 February 2021	Recruiting	Phase 2
		NCT04357366	15 February 2021	Recruiting	Phase 2
		NCT04424056	23 June 2020	Not yet recruiting	Phase 3
		NCT04643678	10 February 2021	Recruiting	Phase 2; Phase 3
		NCT04341584	1 February 2021	Completed	Phase 2
		NCT04362943	28 July 2021	Completed	NA
		NCT04339712	11 January 2021	Completed	Phase 2
Emapalumab	IFN-γ blocking antibody	NCT04324021	14 December 2020	Terminated	Phase 2; Phase 3
Berberine	Cytokine storm	NCT05228626	8 February 2022	Not recruiting yet	Phase 2
		NCT04479202	21 July 2020	Completed	Phase 4
**Canakinumab**	IL-1β	NCT04362813	24 January 2022	Completed	Phase 3
		NCT04348448	16 April 2020	Not yet recruiting	NA
		NCT04365153	15 April 2021	Completed	Phase 2
		NCT04510493	8 September 2021	Completed	Phase 3
Quercetin	TNF-α inhibition	NCT04377789	18 February 2021	Completed	NA
		NCT04468139	13 July 2020	Recruiting	Phase 4
		NCT05037240	8 September 2021	Completed	NA
		NCT05130671	28 January 2022	Completed	NA
		NCT04851821	27 July 2021	Completed	Phase 1
		NCT04853199	28 July 2021	Recruiting	Early phase 1
		NCT04536090	23 August 2021	Not recruiting yet	Phase 2
Berberine	Cytokine storm	NCT05228626	8 February 2022	Not recruiting yet	Phase 2
		NCT04479202	21 July 2020	Completed	Phase 4
Adalimumab	TNF-α	NCT03464136	11 January 2022	Completed	Phase 3

## 6 Therapeutic targets involving viral proteins

As far as blocking the virus by inhibiting viral proteins is concerned, therapeutic targets can be generally divided into two main categories.

✓ Inhibition of viral entry
✓ Inhibition of replication machinery

### 6.1 Inhibition of viral entry

As stated in section 3, SARS-CoV-2 enters the cell through its spike protein by interacting with the host cell receptor (ACE2) and host cell proteases that are involved in the activation of spike. Inhibiting the viral entry may be an efficient way to stop further infection. Multiple strategies are being utilized to block the Spike protein (Dai and Gao, 2020). The most efficient among them is the use of convalescent plasma, which contains anti-SARS-CoV-2 antibodies produced by a recovered COVID-19 patient. Convalescent plasma is being used and tested in a clinical trial (NCT04356534). An anti-spike monoclonal antibody against SARS-CoV-2 is being assessed in a phase 3 clinical trial (NCT04452318).

Blockage of TMRPSS2 is one of the best ways to inhibit the virus entry because this protein is indispensable for the viral pathogenesis. Camostat mesilate and enzalutamide have been reported to inhibit TMPRSS2 *in vitro*, and at present, they are in phase 2 clinical trials (*NCT04455815* and NCT04475601, respectively). Nafamostat mesilate also inhibits TMPRSS2 and is being tested in a phase 3 clinical trial (NCT04390594).

Blocking ACE2 is another choice for blocking the virus entry. It has been demonstrated that ACE2 blockage through derivative peptides can inhibit the infectivity by up to 74% (Yang et al., 2020). Today, several drugs that block ACE2 are in clinical trials. Drugs which inhibit angiotensin receptor have also been evaluated to inhibit viral entry. One of them (losartan) is being evaluated in a phase 4 trial (*NCT04394117* **)**. Another drug—telmisartan—which also blocks ACE2, has been tested in phase 4 clinical trial (*NCT04355936* **)**. Other drugs that block ACE2 have been tested in clinical trials include bromhexine with or without hydroxychloroquine and valsartan (*NCT04355026* and NCT04335786, respectively). Furin inhibitors have been proved to inhibit viral entry and replication, and are further studied for their potential to be used as a therapeutic agent against SARS-CoV-2 (Cheng et al., 2020; Devi et al., 2022).

### 6.2 Inhibition of Replication Machinery

#### 6.2.1 3Cl-Pro

3Cl-pro, also known as Main protease (M-pro), is a chymotrypsin-like protease that is 306 aa long and has a molecular weight of 33.8 kDa. Phylogenetic analysis of 3Cl-pro of SARS-CoV-2 revealed that it shares 96% structural similarity with that of SARS-CoV. 3Cl-pro is first cleaved from the polyprotein between Nsp4 and Nsp6 by autocleavage, and then it cleaves the polyprotein at 11 sites. It also participates in the maturation of the nonstructural proteins and thus gives rise to multiple proteins crucial for viral replication. These proteins include RdRp, helicase, exonuclease, and endoRNase. The fact that this protease is essential for the formation of proteins crucial for viral replication makes it an attractive drug target (Wu et al., 2020).

Lopinavir in combination with ritonavir has been approved by the FDA against HIV. Lopinavir is a protease inhibitor, whereas ritonavir inhibits metabolic inactivation of lopinavir by suppressing cytochrome P450 3A. Thus, ritonavir serves as a metabolic enhancer for lopinavir while lopinavir acts as a protease inhibitor and hence as the main antiviral in this combination (Tobaiqy et al., 2020). As for COVID-19, the main target of lopinavir is 3Cl-pro (Nukoolkarn et al., 2008). Currently, the combination is in a phase 4 trial (NCT04255017). Some toxic and adverse effects of lopinavir/ritonavir have been reported; therefore, these drugs need further research. PF-07321332 is a 3CLpro inhibitor with strong antiviral activity *in vitro* against SARS-CoV-2 and other coronaviruses and it has been tested by *in silico* studies and clinical trial as well (NCT04756531) (Ahmad et al., 2021). Another protease inhibitor that is being evaluated in clinical trials is danoprevir in combination with ritonavir. Danoprevir was approved by China’s FDA in 2018 for the treatment of HCV infection. It targets the NS3 protease in HCV and has a half-maximal inhibitory concentration of 0.29 µM (Chen et al.). It has been tested against COVID-19 in a phase 4 trial (*NCT04345276*). Lately, it is being reported that sitagliptin and daclatasvir target PL-pro, whereas lycorine HCl and nelfinavir mesylate inhibit SARS-CoV-2 M-pro (Narayanan et al., 2022).

PL-pro is the papain-like protease encoded by the *nsp3* gene. It acts on the polyprotein at the N terminus and cleaves it into three nonstructural proteins that are indispensable for the replication cycle. Aside from functioning as the protease, it not only inhibits the innate immune response of the cell by suppressing IFN production but also recognizes the C terminus of ubiquitin and serves as deubiquitinase (Ullrich and Nitsche, 2020).

Therefore, being pivotal for viral replication, PL-pro is an appealing target for therapeutic regimens. Nevertheless, today, there is no FDA-approved drug that targets PL-pro. Possible adverse effects may be a reason for the absence of FDA approval because this drug mimics host deubiquitinases and hence may have a huge number of adverse effects and can be cytotoxic.

#### 6.2.2 RdRp

This is an enzyme that is most crucial for the viral life cycle and is the most conserved enzyme among various viruses including hepatitis C virus, Zika virus, and coronaviruses. Because RdRp structure is conserved among many species, its function and mechanism of action are conserved too. For successful replication of the virus, it first synthesizes RdRp so that positive-sense ssRNA can be produced. Owing to the similarity of structure and function among RdRps of different RNA viruses, it is believed that the broad-spectrum antivirals that act on RdRp may inhibit replication of many different RNA viruses.

RdRp is an essential enzyme catalyzing viral replication by means of the 3′ polyA end of the positive-sense ssRNA as a template to synthesize the complementary strand. Two mechanisms have been proposed to explain the initiation of genome replication. The first one is primer-dependent initiation of synthesis, and the second mechanism is primer-independent synthesis also known as *de novo* synthesis. In case of primer-dependent synthesis, new nucleotides are added to the already present 3′ hydroxyl end provided by the bound oligonucleotide or the protein primer. By contrast, in the case of the *de novo* synthesis, genomic-RNA synthesis is catalyzed by the polymerase itself or by co-factors of the polymerase without any pre-existing 3′ hydroxyl end (Flint et al., 2009). As for SARS-CoV-2, Nsp7 and Nsp8 serve as these co-factors. Nsp8 can synthesize a primer of up to 6 nucleotides (Hillen et al., 2020). The elongation complex consists of 14 bases in the template strand and 11 bases in the primer strand, but for the initiation of the polymerization reaction, the first one or two nucleotides of the primer are very important (Romano et al., 2020).

Targeting RdRp by an antiviral can be effective because stopping this enzyme will inhibit viral replication, and thus the infection cycle will be blocked (Zhao J. et al., 2021; Jukič et al., 2021). Moreover, RdRp has no host cell homolog. Nevertheless, the challenges associated with protein expression, purification, and catalytic activity in *vitro* systems tend to be limitations of RdRp as a target of an antiviral (Zhu et al., 2020).

To date, several drugs that target RdRp have been proposed as COVID-19 therapeutics, and many of them are being tested in clinical trials. At present, remdesivir, favipiravir, and maraviroc are such drugs. Remdesivir, an adenosine analog, has been found to be consistently effective against four families of viruses: Paramyxoviridae, Filoviridae, Pneumoviridae, and Coronaviridae (Pardo et al., 2020). It is reported to be effective against SARS-CoV-2 *in vitro* studies, and at present, it has been tested in phase 3 clinical trial (NCT04409262).

Another promising drug is favipiravir. It is a guanine analog that inhibits RdRp. It was initially approved by the FDA in 2014 and is used against influenza A and B. It has been proposed as a drug against influenza strains that are resistant to neuraminidase inhibitors (Li H. et al., 2020). Favipiravir was recently tested against COVID-19 and was found effective, with an excellent half-maximal effective concentration of 61.88 µM. It has been tested in phase 3 clinical trial (*NCT04464408*).

Ribavirin has been employed in combination with IFN-α against HCV (Hadziyannis et al., 2004). It is a guanosine analog and has been found to exert broad-spectrum antiviral action against various DNA and RNA viruses (Te et al., 2007). Today, ribavirin in combination with IFN-β-1b is being assessed in a phase 2 clinical trial (NCT04494399). Besides, ribavirin is being tested in combination with sofosbuvir in another clinical study currently in phase 3 trial (*NCT04460443* **).**

### 6.3 Accessory proteins

#### 6.3.1 Exonuclease

Some of the most widely used antivirals are the nucleotide analogs that block viral replication by getting incorporated into the elongated genome, consequently leading to termination of the genome synthesis, thereby inhibiting the viral replication cycle. This approach is particularly useful against RNA viruses because RNA polymerase does not possess a proofreading activity. This drawback makes RNA viruses more susceptible to mutations and to nucleotide-based inhibitors (Modrow et al., 2013). Nonetheless, coronaviruses are an exception in this context because they possess exonuclease and endonuclease. The N terminus of Nsp14 serves as the exonuclease. A nucleotide analog incorporated in the growing strand is readily removed from the strand. Hence, the virus continues its replication without any hinderance (Gurung, 2020). Moreover, guanine-N7 methyltransferase, which is involved in the capping of SARS-CoV-2 is also being investigated for targeting in order to interfere with viral pathogenesis (Selvaraj et al., 2021). In an *in* silico study, Saquinavir, Hypericin, Baicalein and Bromocriptine have been found to interact with Nsp14 (Liu et al., 2021a). Therefore, Nsp14 can serve as a promising target, particularly when RdRp is being suppressed with nucleotide analogs.

#### 6.3.2 Helicase

Helicases are multifunctional proteins of Coronaviridae and have been conserved over the course of evolution. The helicase of SARS-CoV-2 belongs to helicase superfamily 1 (Shu et al., 2020). It contains three main domains: an N-terminal domain, metal-binding domain, and helicase domain. Helicase hydrolyzes nucleotides of DNA as well as RNA to unwind the helix in the 5′-to-3′ direction and is a crucial component of viral replication machinery (Wu et al., 2020). Therefore, this enzyme has been suggested as an effective druggable target. Some authors reported that vapreotide and atazanavir are candidate drugs against the helicase of SARS-CoV-2 (Francis Borgio et al., 2020). Atazanavir is currently being evaluated in a clinical trial against COVID-19 (NCT04468087).

#### 6.3.3 Virulence Factors

Apart from participating in replication, viral proteins interfere with the host immune system and either antagonize or enhance the immune response depending upon the context. Such proteins can also be a target for drug design.

Nsp1, also known as the leader peptide, is encoded by the first nonstructural-protein gene in the SARS-CoV-2 genome. Nsp1 of SARS-CoV and that of SARS-CoV-2 are 100% similar in amino acid sequence. Some authors reported that Nsp1 of SARS-CoV inhibits IFN-β expression; furthermore, it induces the degradation of host endogenous mRNAs (Kamitani et al., 2006). It has also been observed that SARS-CoV-2 ORF9b interacts with TOM70 and inhibits IFN-β production, and hence no antiviral response from the host machinery is induced (Jiang et al., 2020). Moreover, viral protein ORF9b participates in immune evasion too, by influencing the interaction of MAVS with TOM70 (Konno et al., 2020; Ribero et al., 2020).

## 7 Concluding Remarks

SARS-CoV-2 has been causing great harm since the beginning of the pandemic. As reviewed above, multiple innate-immunity receptors play a part in the pathogenicity of SARS-CoV-2. Despite the availability of vaccines and drugs, this virus still poses a great danger to humanity. Although it is not very common for RNA viruses to possess exonuclease activity, SARS-CoV-2 has the exonuclease activity as it belongs to *Nidovirales* order. Despite the exonuclease activity, the emergence of multiple variants has made it clear that the viral proteins can keep on mutating (thereby making the available drugs inefficacious against SARS-CoV-2). This phenomenon points to the importance of exploring the therapeutic potential of human endogenous immune receptors against COVID-19. Innate immune system is body’s first line of defense against any invading pathogen. A wide variety of innate-immunity receptors including TLRs, RLRs, CLRs, and their accessory signaling molecules play an important role in virus sensing and in the development of an optimal immune response. Once the viral components are detected by the cellular innate immune receptors, the body mounts an optimum immune response against SARS-CoV-2. The key features of the antiviral immune response include interferon generation, cytokine production and cell death. However, viral proteins interfere the immune response and create trouble for the body by hyperactivating the immune system which leads to cytokine storm. As a result of cytokine storm and other co-morbidities, the disease complications and severity increases, thus causing death of the host.

The present review has summarized current knowledge about innate-immunity receptors and about signaling molecules in relation to COVID-19 pathophysiology as well as the possibility of their use as druggable targets. Our review also summarizes the knowledge about currently available drugs as well as the drugs under clinical trials which are be being tested these days. Various drugs which target immune receptors and other signaling molecules have also been summarized in the review. Antibodies targeting cytokines and their receptors, including Canakinumab, Anakinra, Emapalumab, Sarilumab, and Tocilizumab, are under clinical trials. Although the success rate is relatively low when it comes to the application of innate-immunity receptors for the therapeutic purposes, the high mutation rate of RNA viruses makes these receptors a promising option.
